# An Intelligent Motor Imagery Detection System Using Electroencephalography with Adaptive Wavelets

**DOI:** 10.3390/s22218128

**Published:** 2022-10-24

**Authors:** Smith K. Khare, Nikhil Gaikwad, Neeraj Dhanraj Bokde

**Affiliations:** 1 Department of Electrical & Computer Engineering, Aarhus University, 8000 Aarhus, Denmark; 2 Center for Quantitative Genetics and Genomics, Aarhus University, 8000 Aarhus, Denmark

**Keywords:** electroencephalogram signals, intelligent system, evolutionary optimization algorithms, adaptive wavelets, Laplacian score, support vector classifiers

## Abstract

Classification of motor imagery (MI) tasks provides a robust solution for specially-abled people to connect with the milieu for brain-computer interface. Precise selection of uniform tuning parameters of tunable Q wavelet transform (TQWT) for electroencephalography (EEG) signals is arduous. Therefore, this paper proposes robust TQWT for automatically selecting optimum tuning parameters to decompose non-stationary EEG signals accurately. Three evolutionary optimization algorithms are explored for automating the tuning parameters of robust TQWT. The fitness function of the mean square error of decomposition is used. This paper also exploits channel selection using a Laplacian score for dominant channel selection. Important features elicited from sub-bands of robust TQWT are classified using different kernels of the least square support vector machine classifier. The radial basis function kernel has provided the highest accuracy of 99.78%, proving that the proposed method is superior to other state-of-the-art using the same database.

## 1. Introduction

The recent increase in Brain-computer interface (BCI) has attracted researchers to develop solutions for computers and other devices with the aid of brain activities. BCI manipulates the brain’s electrical activities to help the specially-abled carry out their routine tasks. The cognitive activities accessed through electroencephalogram (EEG) signals provide an intelligent solution to assist specially-abled individuals to accomplish body movement just by mere imagination without any external support is motor imagery (MI) classification [1]. Non-invasive nature and wide exposure of EEG signals for various neurological activities makes it most viable for modelling BCI systems [2,3,4,5,6].

Several techniques using EEG signals exploring time, frequency and time-frequency (TF) domain computation have been developed to detect MI tasks. Separation of MI tasks by distinction sensitive learning vector quantization based adaptive autoregression model has been proposed in [7]. Welch spectral analysis method based on fast Fourier transform (FFT) with decision tree classifier has been used for discrimination of brain activities [8]. Regularized common spatial patterns (R-CSP) [9], filtered regularized CSP (FRCSP) [10], weighted and regularized CSP [11], CSP with particle swarm optimization (PSO) [12], optimization of filterbanks and time window within CSP [13] and adaptive removal of artifacts within CSP [14] have been explored for the MI classification. Cross-correlation (CC) and support vector machine with least square (LS-SVM) has been used in [15] for bifurcation of MI tasks. Cross-correlation [16] and modified cross-correlation (M-CC) [17] with logistic regression (LR) have been proposed for identification of MI. The utility of optimal allocation (OA) algorithm using Naive Bayes (NB) and LS-SVM have been used in MI recognition [18,19]. Power spectral density, phase lock value (PLV) with CC [20], and PLV with spectral coherence [21] have been proposed for MI detection.

Wavelet transform (WT) and Wilcoxon statistical method with tabu fuzzy standards have been used in [22]. Isolation of MI has been accomplished using machine learning techniques combined with discrete WT (DWT) [23,24], rational dilation WT (RDWT) [25], and tunable Q WT (TQWT) [26]. Empirical mode decomposition (EMD) with Hilbert transform [27] based on AM-FM intrinsic mode functions has also been used for recognizing MI tasks. Several TF analysis has also been proposed for MI classification. Classification of MI tasks using TF analysis filtered based FFT with long-short term memory [28], WT [29], and continuous WT (CWT) [30] with convolutional neural networks (CNN) have been proposed. Multivariate EMD with Gaussian SVM has been used for MI identification [31]. In addition to this, Z-score [32], multiscale principal component analysis (MSPCA) with wavelet packet decomposition (WPD) [33], clustering technique (CT) [34], adaptive space-time-frequency analysis [35] and comprehensive spectral methods [36] have been proposed for segregations of MI tasks. An automated form of variational mode decomposition combined with extreme learning machine classifier has been also used to detect MI tasks [37]. In [38], the authors explored hybridization of MSPCA combined with WPD to extract hidden information from EEG using subbands. The utility of flexible analytic wavelet transform (FAWT) has been used to extract and classify the evaluated time-frequency features from the subbands using linear discriminant analysis classifier [39]. The time-series analysis using a CNN module has been explored to detect MI tasks [40]. The authors in [41] explored multiscale Siamese CNN with cross-channel fusion to detect MI. The classification of MI has been accomplished using multi-branch hybrid neural network [42]. Efficient and accurate modelling of BCI systems is only possible with proper selection of channels, decomposition technique, machine learning or deep learning framework, and evaluation of performance indices [43]. Most of the signal decomposition methods used in the literature use an empirical selection of fixed basis functions. The choice of fixed basis function for non-stationary EEG signals may not properly decompose EEG signals. This inspires us to present adaptive tuning parameters selection for fruitful EEG analysis. This methodology proposes robust TQWT using evolutionary optimization algorithms (EOA) for quintessential tuning parameters and Laplacian score for channel selection. The novelty of the method is as follows:

- Exploring evolutionary optimization for adaptive selection of proper basis function;
- Use of optimum decomposition level to decompose the signal into sub-bands;
- Exploring the utility of Laplacian score for selecting proper channels to reduce computational complexity.

The automated and adaptive TQWT has been used to various physiological conditions (sleep, emotions, and drowsiness) [44,45,46] and neurological disorders (schizophrenia and Parkison’s disease) [47,48,49]. The paper is structured as datasets, preprocessing, robust TQWT, feature extraction, channel selection, and classification are described in Section 2. Section 3 explains the results of computation of the explored method, discussion with state-of-the-art techniques (SOTA) in Section 4. The gist of the developed technique is presented in Section 5.

## 2. Methodology

### 2.1. Data-Set

**Figure 1:**
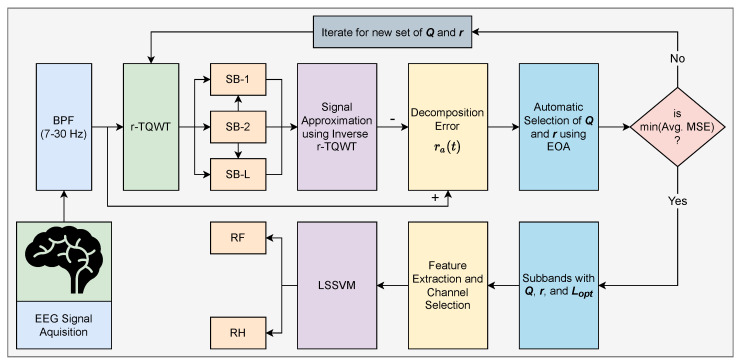
Workflow of proposed robust TQWT framework with channel selection.

The proposed method uses the IV-a dataset of BCI competition-III [50]. MI-tasks of five healthy subjects (**aa**, **al**, **av**, **aw**, **ay**) sat on comfortable chairs have been recorded. Subjects performed the MI tasks of right foot (RF) (task1) and right hand (RH) (task2). According to the international 10–20 system, one hundred eighteen electrodes are used to acquire the EEG recordings of MI-related tasks. For intimation of MI tasks, subjects are shown with visual cues of 3.5 s before acquiring EEG signals [51]. For each subject, a total of 280 trials are taken. Each task has 140 trials equally. Each subject consists of different sizes of training and testing sets. Out of 280 trials, for subjects **aa**, **al**, **av**, **aw**, **ay** training set composed of 168, 224, 84, 56 and 28 trials and remaining used for testing. The actual sampling rate in recording EEG signals is 1000 Hz, later downsampling at 100 Hz. The stepwise working of the proposed method is shown in Figure 1.

### 2.2. Pre-Processing

**Figure 2:**
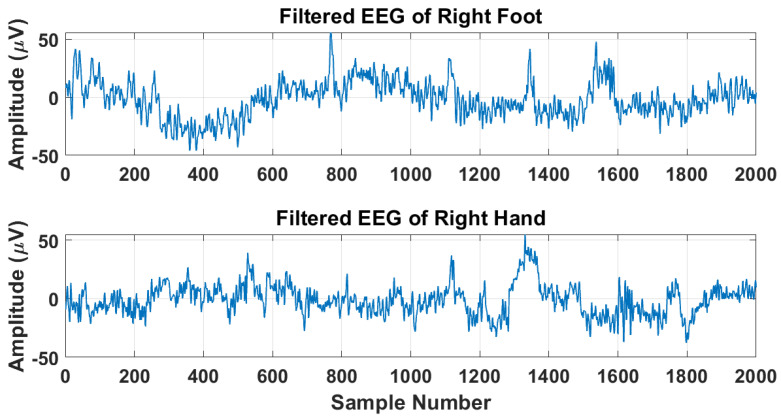
Examples of EEG signals for RF and RH brain activity.

For BCI, two types of oscillations play a vital role, Rolandic mu and central beta lying in the frequency range of 7-30 Hz [52]. These oscillations originate in the sensorimotor cortex. Hence the EEG signals are bandpass through a sixth-order Butterworth IIR filter in the frequency range of 7-30 Hz. After the BPF, the filtered data of RF and RH has been segmented into 250 and 200 epochs of length 2000 samples each for every channel for further processing. The RF and RH EEG signals are shown in Figure 2, respectively. It is evident from the Figure that both filtered EEG signals of RF and RH have the same amplitude range, making it difficult to classify MI tasks.

### 2.3. Robust Tunable Q Wavelet Transform (r-TQWT)

A TQWT is a parametrized discrete-time WT with a tunable quality factor. To decompose the signal into subbands (SBs) using TQWT requires quality factor (*Q*), redundancy rate (*r*) and levels of decomposition (*L*) as a basis function. Decomposing a signal with *L* level results in the generation of one lowpass SB (LPS) and *L* highpass SBs (HPS). TQWT can tune the *Q* as per the oscillatory behavior of signals. The higher quality factor is recommended for better frequency resolution and a higher redundancy rate for the well-localized time domain. After the *L* level decomposition of signal, LPS $V_{0}^{L}\left(\omega \right)$ and HPS $V_{1}^{L}\left(\omega \right)$ are represented by [53]

(1) $$V_{0}^{L}\left(\omega \right)=\left\{\begin{array}{cc} \prod_{l=0}^{L-1}V_{0}\left(\frac{\omega }{\alpha^{m}}\right), & \left|\omega \right|\leq \alpha^{L}\pi \\ 0, & \alpha^{L}\pi <\left|\omega \right|\leq \pi \end{array}\right.$$

(2) $$V_{1}^{L}\left(\omega \right)=\left\{\begin{array}{cc} V_{1}\left(\frac{\omega }{\alpha^{L-1}}\right)\prod_{m=0}^{L-2}V_{0}\left(\frac{\omega }{\alpha^{m}}\right), & \left(1-\beta \right)\alpha^{L-1}\pi \\ \; & \leq \left|\omega \right|\leq \alpha^{L-1}\pi \\ 0, & \omega \in [-\pi ,\pi ] \end{array}\right.$$

With the tuning property of *Q* and *r* following the highly oscillatory nature of EEG signals, TQWT has gained wide acceptance in signal processing. The experiential selection of fixed tuning parameters may lead to the improper decomposition of EEG signals and the loss of some critical information. Setting a common for different EEG signals can lead to a higher decomposition error. The non-stationary nature of EEG signals requires the different values of tuning parameters for every set EEG signal for accurate reconstruction and better decomposition. This inspires us to propose the robust TQWT for selecting appropriate tuning parameters with the help of EOAs using the fitness function of mean square error (MSE) of decomposition. To resolve an issue of uniform tuning parameters, robust TQWT is proposed to automate the selection of *Q* and *r* using EOAs. The steps for robust TQWT are described in Algorithm 1. The quality factor of TQWT, which must be higher for signals with higher oscillations, is represented by

(3) $$Quality\;Factor\;\left(Q\right)=\frac{2-\beta }{\beta }$$

**Algorithm 1** Robust TQWT1:Select the *Q* and *r* initially to 1.2:Calculate $L_{max}$ from the values of *Q* and *r*.3:Decompose EEGs with *Q*, *r* and $L_{max}$.4:Get the approximated original signal by inverse robust TQWT using *Q*, *r* and *M* (No. of time samples).5:Compute the MSE of decomposition error $w_{e}\left(t\right)$.6:**while***($min(w_{e}\left(t\right))$)***do**7:** if** (*w_e_*(t)== min) **then**8:  Optimum tuning parameters ($Q_{{opt}_{i}}$ and $r_{{opt}_{i}}$).9: **else**10:  Iterate for next values of *Q* and *r*.11: **end if**12:**end while**13:Obtain the maximum decomposition levels $L_{{max}_{i}}$ from $Q_{{opt}_{i}}$ and $r_{{opt}_{i}}$.14:Evaluate the optimum decomposition levels from *L_max_*.15:Obtain the SBs of EEG signals using the optimum tuning parameters.
where $Q_{{opt}_{i}}$, $L_{{max}_{i}}$ and $r_{{opt}_{i}}$ are optimum tuning parameters for signal *i*.


The time-domain response of the signal is well-localized if *r* is ≥ 3. The redundancy rate is defined as

(4) $$r=\frac{\beta }{1-\alpha }$$

Maximum levels of decomposition in TQWT is denoted by

(5) $$L_{max}=|\frac{\log\left(\frac{M}{4*(Q+1)}\right)}{\log\left(\frac{Q+1}{Q+1-\frac{2}{r}}\right)}|$$

An EEG signal is represented as a combination of approximated and error signal that can be expressed as

(6) $$f\left(t\right)=\hat{f}\left(t\right)+d_{e}\left(t\right)$$

where f(t) is the original signal in time domain, $\hat{f}\left(t\right)$ denotes approximated signal evaluated by inverse TQWT, and decomposition error is $d_{e}\left(t\right)$. To reconstruct the decomposed EEG signal with minimum decomposition error, mean square error is chosen as an fitness function of EOA represented by

(7) $$w_{e}\left(t\right)=\int_{-\infty }^{+\infty }{\left[f\left(t\right)-\hat{f}\left(t\right)\right]}^{2}dt$$

**Figure 3:**
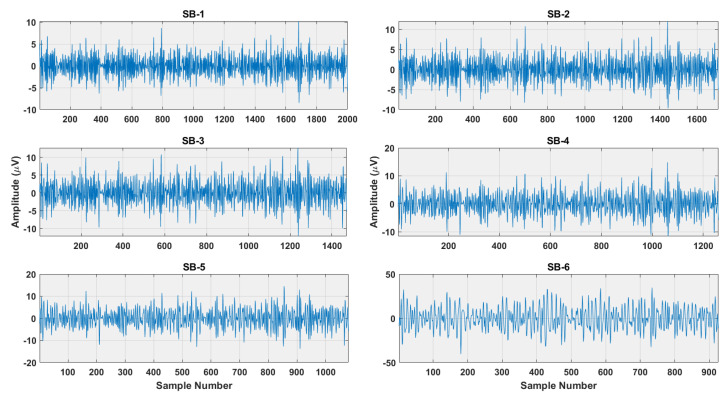
The subbands of RF brain activity obtained after r-TQWT decomposition.

**Figure 4:**
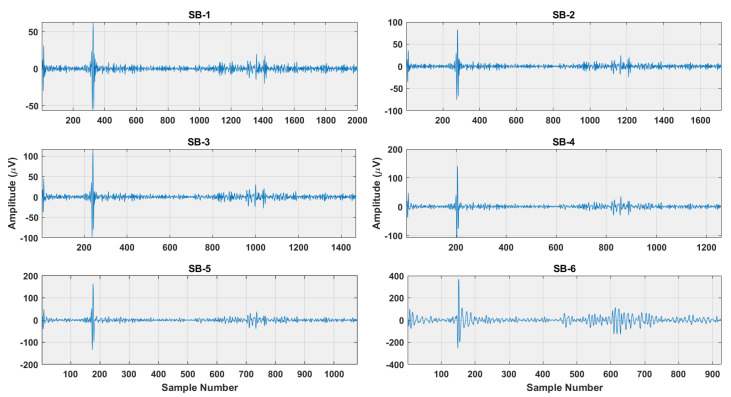
The subbands of RH brain activity obtained after r-TQWT decomposition.

Obtaining the minimum decomposition error of EEG signals by manually selecting the optimum tuning parameters is laborious. To overcome this, heuristic optimization techniques, namely PSO, ABC, and CS, are used to automate the tuning parameters. Details of these optimization algorithms are available in [54]. Optimization algorithms evaluate the fittest solution by repetitive iterations using several search agents. PSO mimics the behaviour of bird flocks and fish schooling to get the optimal global solution. The fittest solution formulas of PSO are expressed by

(8) $$\begin{array}{cc} v_{i}^{n+1} & =Y[v_{i}^{n}+\varphi \left(a_{1}.\left(p_{i}^{n}-x_{i}^{n}\right)+a_{2}.\left(g_{i}^{n}-x_{i}^{n}\right)\right)]\\ x_{i}^{n+1} & =x_{i}^{n}+v_{i} \end{array}$$

where Y is the constriction factor to increase the chances of convergence, $p_{i}$ and $g_{i}$ are the personal and global best. $a_{1}$ and $a_{2}$ are the personal and social cognitive factors, and φ is the random number in the range 0 to 1. The ABC algorithm copies the honey bees’ intelligent behaviour in search of nectar. The update equation to find the optimal solution is given by

(9) $$\begin{array}{cc} v_{i} & =x_{i}+\kappa_{i}\left(x_{i}-x_{r}\right)\\ h_{i}\left(\vec{x_{i}}\right) & =\left\{\begin{array}{cc} \frac{1}{1+f_{i}\left(\vec{x_{i}}\right)} & f_{i}\left(\vec{x_{i}}\right)\geq 0\\ 1+abs(f_{i}\left(\vec{x_{i}}\right)), & f_{i}\left(\vec{x_{i}}\right)<0 \end{array}\right.\\ p_{s_{i}} & =\frac{h_{i}\left(\vec{x_{i}}\right)}{\sum_{i=1}^{FN}h_{i}\left(\vec{x_{i}}\right)} \end{array}$$

where $v_{i}$ and $x_{i}$ are the $i^{th}$ particle velocity and position, $x_{r}$ is the randomly selected food source, fitness function is denoted by $h_{i}$, κ is random number in the range {−1, 1} both are inclusive. $f_{i}$ is the value of fitness function for solution $\vec{x_{i}}$, probability is denoted by $p_{s_{i}}$ and number of food sources are FN. The cuckoo search algorithm impersonates the cuckoo bird species’ broot parasite and Levy flight property. Following the Levy flight, the new solution of CS is generated by

(10) $$\begin{array}{c} x_{i}\left(n+1\right)=x_{i}\left(n\right)+\delta ⊗Levy\left(\beta \right)\\ Levy∼\mu =n^{-1-\beta }\;\;\;\;\;\;\left(0<\beta <2\right) \end{array}$$

where Levy is the Levy distribution, δ is the step size mostly equal to 1, and ⊗ is the matrix multiplication. Once the optimum *Q* and *r* are obtained from EOA, the maximum decomposition level is calculated from Equation (5). To maintain the uniformity among all the decomposed EEG signals, the optimum level of the decomposition $L_{opt}$ is evaluated. The equation of the $L_{opt}$ is defined by

(11) $$L_{opt}=\frac{1}{P}\sum_{c=1}^{C}\frac{1}{C}\sum_{p=1}^{P}L_{max}$$

where *P* is the number of signals belonging to each class and *C* are the number of classes. The examples of subbands obtained after r-TQWT decomposition for RF and RH brain activities are shown in Figure 3 and Figure 4. It is seen from the Figures that subbands of RH and RF have different amplitude ranges, which depicts their better discrimination ability. Thus, the decomposition of r-TQWT extracts insight information of the EEG signals. Therefore, the idea of r-TQWT is justified for detecting brain activities.

### 2.4. Features Extraction and Channel Selection

Features represent the statistical measures of the decomposed EEG signals. To discriminate the motor imagery tasks, five statistical parameters are evaluated with details as follow.

#### 2.4.1. Hurst Exponent (HE)

HE measures long-term memory of time series. HE quantifies the relative tendency of time series either regress sharply to cluster in a direction or to the mean.

(12) $$HE=E\left[\frac{r(J)}{\sigma (J)}\right]$$

where *E* is the expected value, *r* is the range, *J* are the number of samples in each signal and σ is the standard deviation.

#### 2.4.2. Modified Mean Absolute value1 (MAV1)

MAV1 [55] is the extension of mean absolute value with assignment of weighted window function $w_{j}$ for improving the robustness.

(13) $$\begin{array}{cc} MAV1 & =\frac{1}{J}\sum_{j=1}^{J}w_{j}\left|y_{j}\right|\\ w_{j} & =\left\{\begin{array}{cc} 1, & 0.25J\leq j\leq 0.75J\\ 0.5, & otherwise \end{array}\right. \end{array}$$

#### 2.4.3. Difference Absolute Standard Deviation Value (DASDV)

DASDV [55] is representing the standard deviation value of the wavelength.

(14) $$DASDV=\sqrt{\frac{1}{J-1}\sum_{j=1}^{J-1}{\left(y_{j+1}-y_{j}\right)}^{2}}$$

#### 2.4.4. Log Energy Entropy (LEE)

LEE [56] is a measure of the complexity of the EEG signals.

(15) $$LEE=-\sum_{j=0}^{J-1}(\log_{2}{\left(p_{j}\left(y\right)\right)}^{2}$$

where $p_{j}$ is the probability of occurance of y.

#### 2.4.5. Variance

Variance measure the variation in the data points of the data defined by

(16) $$Variance\left(\sigma^{2}\right)=\frac{\sum {\left[y-\mu \right]}^{2}}{J}$$

The features elicited from the filtered robust TQWT decomposed SBs are computed for 118 channels. To reduce the complexity of the BCI system, instead of exploring all the channels for classification, the adequacy of the single EEG channel is used in this framework. Laplacian score (LS), based on Laplacian Eigenmaps and locality preserving projection, is explored for selecting appropriate channels. The Laplacian score for the $k^{th}$ feature is defined as [57].

(17) $$\begin{array}{c} L_{k}=\frac{{\tilde{F_{k}}}^{T}L\tilde{F_{k}}}{{\tilde{F_{k}}}^{T}D\tilde{F_{k}}}\\ \tilde{F_{k}}=F_{k}-\frac{F_{k}^{T}D1}{1^{T}D1}1\\ S_{ij}=exp^{\frac{-\left|\right|y_{i}-y_{j}{\left|\right|}^{2}}{t}} \end{array}$$

where $F_{k}$ is the $k^{th}$ feature matrix, *T* is the transform, $1={\left[1,1,.....1\right]}^{T}$, *D* = diag(*S*1) and the Laplacian graph L=S−D. *i* and *j* correspond to nodes $y_{i}$ and $y_{j}$, while *t* is the constant, respectively.

### 2.5. Classifiers

Statistically, significant features are classified using classification techniques. This methodology uses the LS-SVM classifier to discriminate against the RH and RF activities. LS-SVM involves the formulation of inequality constraints that transform non-linear problems into linear equations with a rigid regression cost function. For a training set of data length of *N* points ${\left\{z_{i},y_{i}\right\}}_{i=1}^{N}$ the decision function of the LS-SVM classifier [58] is defined as

(18) $$z\left(y\right)=sign\left\{\sum_{i=1}^{N}a_{i}z_{i}\Psi \left(y,y_{i}\right)+\beta \right\}$$

where $y_{i}\in I\;R^{n}$ is the $i^{th}$ input feature vector and $z_{i}\in I\;R$ is the $i^{th}$ output pattern. $a_{i}$ is the positive real coefficient, β is the real constant biasing term, $\Psi (y,y_{i})$ is the kernel function. Five kernels namely linear, polynomial, Mexican hat (MHW), Morlet (MW), and Radial basis function (RBF) have been used in this framework to test the compatibility of the proposed method. The details of these kernels are available in [59]. The linear kernel is expressed as

(19) $$\Psi \left(y,y_{i}\right)=\left(y_{i}^{T}y\right)$$

The polynomial kernel can be defined as

(20) $$\Psi \left(y,y_{i}\right)={\left(y_{i}^{T}y+1\right)}^{l}$$

The equation of MHW kernel is denoted as

(21) $$\Psi \left(y,y_{i}\right)=\prod_{i=1}^{N}1-\left(\frac{{\left(y-y_{i}\right)}^{2}}{a_{i}^{2}}\right).exp^{\frac{-||y-y_{i}{\left|\right|}^{2}}{2a_{i}^{2}}}$$

MW kernel is represented as

(22) $$\Psi \left(y,y_{i}\right)=\prod_{i=1}^{N}cos\left(\omega_{0}\frac{\left(y-y_{i}\right)}{a_{i}}\right).exp^{\frac{-||y-y_{i}{\left|\right|}^{2}}{2a_{i}^{2}}}$$

The RBF kernel is defined by

(23) $$\Psi \left(y,y_{i}\right)=exp^{\frac{-||y-y_{i}{\left|\right|}^{2}}{2\sigma^{2}}}$$

where *l* is the degree of polynomial kernel, σ is the RBF kernel width, $\omega_{0}$ and *a* are the parameters of MHW and MW kernels.

## 3. Results

The selection of fixed basis functions for decomposing non-stationary EEG signals may result in information loss. Setting the tuning parameters of TQWT may not yield the desired results, leading to higher misclassification. The method proposed in this paper uses the automatic selection of tuning parameters to decompose EEG signals using EOA accurately. PSO, ABC, and CS optimizations are used for self-selection of *Q*, *r* and $L_{opt}$ to excerpt the time-domain features from the SBs of decomposed signals. To maintain the effectiveness of the proposed method number of iterations (200) and search agents (50) is kept uniform. To choose the tuning parameters adaptively for every EEG signal, the fitness function of the MSE of the reconstructed and the original signal is considered.

The decomposition error obtained for robust TQWT using EOA is shown in Table 1. The average MSE using EOA is minimum in the case of CS algorithm with 2.548 $\times \;10^{-05}$ for RF and 2.352 $\times \;10^{-05}$ for RH while it is maximum for PSO producing the error as 7.822 $\times \;10^{-05}$ and 5.997 $\times \;10^{-05}$ for RF and RH, respectively. The least average MSE is obtained for the cuckoo search algorithm, which inspires us to use the optimum parameters of this algorithm over other EOA. $L_{max}$ evaluated from *Q* and *r* is used to compute $L_{opt}$ from Equation (11) is obtained as 5. These optimum *Q*, *r* and $L_{opt}$ are employed to obtain the SBs of EEG.

Features from 118 electrodes are elicited from decomposed SBs. Employing features of 118 electrodes for classifying MI tasks increases the system’s complexity. Therefore, the Laplacian feature score is evaluated to select the substantial channel for performance evaluation. The Laplacian score of the five time-domain features is shown in Table 2. Out of 118 channels, LS of the best performing seven channels is shown in the table. Features of a channel with the highest Laplacian score are considered significant for classification. LS for four features, MAV1, LEE, variance, and HE, produce the highest score for channel-1, while DASDV provides the best score for channel-9. Results of Table 2 prove the superiority of channel-1; hence features of this channel are taken into consideration for further evaluation. Kruskal Wallis (KW) analysis is applied to the extracted features of channel-1 to check the discrimination ability of the features. KW test is a non-parameterized analysis of variance that gives the chi(p) probability. The probabilistic values of *p* < 0.05 are considered to be significant. Table 3 represents the probabilistic values of various features of the SBs on channel-1. As observed from the table, five features, MAV1, LEE, DASDV, variance, and HE, show the compelling discrimination ability for all the SBs and motivate us to use these feature sets for the segregation of the MI tasks.

Five kernels of LS-SVM classifiers are employed in this method to evaluate the classification accuracy of different SBs of channel-1. Table 4 indicates the accuracy (ACC) score of SBs using the LS-SVM classification technique. Table 4 shows that the worst-performing is the linear kernel, while the RBF kernel gives the best performance in all the SBs. SB5 is most superior, while SB6 is most inferior in the case of the linear kernel with a classification accuracy of 73.56% and 61.56%. The highest accuracy score provided by polynomial and Morlet is in SB4, providing an ACC of 89.78% and 92.44%, while the ACC of 97.56% is recorded highest in SB3 for the Mexican hat kernel. RBF kernel is the best performing kernel among all with ACC of 95.56%, 98.22%, 96%, 95.78%, 99.78% and 72.44% for SB1, SB2, SB3, SB4, SB5 and SB6, respectively.

**Figure 5:**
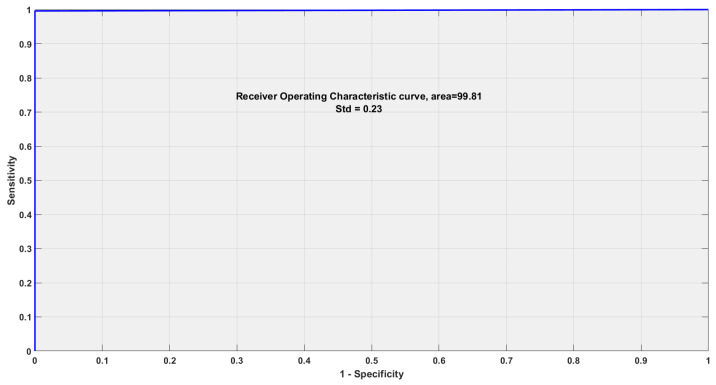
Receiver operating characteristics and the area under the curve obtained for binary classification of RH and RF activities.

As the RBF kernel overperforms other kernels, it is used to evaluate the performance parameters of channel-1. Table 5 shows the results of the performance parameters evaluated on all the SBs of channel 1. Five performance parameters are evaluated to test the dominance of the proposed methodology, viz. ACC, sensitivity (SEN), specificity (SPE), F-1 score and Matthews correlation coefficient (MCC), respectively. As observed from the table, SB5 gives the best results, while SB-6 is the worst performing SB. ACC of 99.78%, SEN and SPE are 99.60% and 100%, F-1 score is noted 0.998 while MCC 99.55%is marked as best scores for SB5. Worst values of ACC, SEN, SPE, F-1 score and MCC is obtained in SB6 as 72.44%, 75%, 69.19%, 0.752% and 44.14% respectively. The binary classification ability of the model is evaluated using the analysis of receiver operating characteristics and area under the curve, as shown in Figure 5. Our model has obtained the area under the curve of 99.81% with a minimal standard deviation depicting its binary classification ability. The confusion matrix of RF and RH is indicated in Table 6. The confusion matrix of RF is given 100% correct and 0% misclassification, while RH provides an incorrect classification of 0.5% and 99.5% of correct classification.

## 4. Discussion

The proposed framework is compared with the existing SOTA using the same database for its effectiveness. The performance of the proposed method is compared with 15 SOTA techniques considering the number of channels and features they have used, as shown in Table 7. Ince et al. [35] used the class separability (CT) method with SVM using three channels and eight features with 96% accuracy. Different versions of CSP have also been employed by Park et al. [10], and Lu et al. [9] to detect the MI tasks with an accuracy mark of 86.23% and 88.32%. Siuly et al. explored cross-correlation based signal processing [15,16,17] and optimal allocation [18,19] for recognition of MI tasks. The highest accuracy achieved is 96.62% while using OA and LS-SVM. Zhang et al. [32] used Z-score claimed 81.1% accuracy using linear discriminant analysis (LDA); the ACC of 96.1% was achieved by Verma et al. [33] using DWT. Kevric et al. [23] used MSPCA, WPD and higher-order statistics (HOS), claiming an ACC of 92.8%. Taran et al. [26,27] used EMD, and TQWT to discriminate brain activities and achieved an ACC of 96.89% and 97.56% using LS-SVM, respectively. Taran et al. [25] used RDWT and quadratic SVM classifier combining 10 channels and five features to obtain an ACC of 99.11%. Recently proposed MI tasks classification method using CWT and CNN by Chaudhary et al. [30] achieved an ACC of 99.35%. Subasi and Qaisar [38] developed a hybrid model combing hybridization of MSPCA and WPD to extract the SB. Six statistical measures extracted from the SB have been classified using ensemble classifier techniques. Their model yielded the highest accuracy of 98.69% using an RF classifier. The proposed method uses single-channel selection by Laplacian score and four time-domain features for classification of MI tasks using RBF kernel to achieve an ACC of 99.78% which is higher than all the SOTA using the same database.

The developed model has following advantages:

- The model is robust (developed using a 10-fold cross-validation technique).
- The developed model is simple (requiring only a single channel and five features to compute model performance).
- The model is data-driven (does not require selection of basis).

However, the model suffers some limitations which are as follows:

- The model is tested on a single EEG dataset with only five subjects.
- The model does not provide explainability for the predictions.

## 5. Conclusions

This method explores the utility of automatic selection of optimum tuning parameters of robust TQWT using evolutionary optimization algorithms. The adaptive selection of optimum tuning parameters is accomplished using PSO, ABC and CS algorithms. CS provides the fittest solution for the objective function; hence, the tuning parameters of CS are used to evaluate optimum decomposition levels and decompose the signal with these parameters. This method exploits the LS for selecting a dominant channel for segregating MI tasks using the LS-SVM classifier. The proposed method obtained an ACC of 99.78% which is higher than all other methods using the same database. The method proposed in this paper can be employed in a real-time BCI system to recognise MI tasks with lowered complexity as it uses only a single channel with a lower number of features. 

## Figures and Tables

**Table 1:** The average MSE obtained for the EEGs of RF and RH brain activities using three EOA.

EOA	Average MSE for the EEGs of RF and RH	
	RF	RH
PSO	7.822 × $10^{-05}$	5.997 × $10^{-05}$
ABC	4.356 × $10^{-05}$	2.929 × $10^{-05}$
CS	2.548 × $10^{-05}$	2.352 × $10^{-05}$

**Table 2:** Laplacian score of the features for the seven best channels.

Features	CH1	CH2	CH3	CH5	CH6	CH9	CH11
MAV1	0.990	0.549	0.847	0.804	0.892	0.241	0.137
LEE	0.914	0.525	0.711	0.356	0.899	0.468	0.401
DASDV	0.677	0.466	0.491	0.660	0.682	0.890	0.810
Variance	0.076	0.008	0.013	0.015	0.054	0.015	0.033
HE	0.911	0.706	0.621	0.756	0.825	0.745	0.730

**Table 3:** Probability of chi (p)-Values of channel 1 SBs.

SB	HE	MAV1	DASDV	LEE	Variance
SB1	6.7 × $10^{-03}$	1.9 × $10^{-03}$	2.3 × $10^{-05}$	7.3 × $10^{-55}$	2.0 × $10^{-04}$
SB2	1.9 × $10^{-04}$	2.3 × $10^{-03}$	8.7 × $10^{-06}$	5.6 × $10^{-50}$	3.2 × $10^{-04}$
SB3	1.2 × $10^{-05}$	8.3 × $10^{-03}$	6.1 × $10^{-06}$	9.2 × $10^{-45}$	7.5 × $10^{-04}$
SB4	4.1 × $10^{-05}$	2.5 × $10^{-02}$	7.6 × $10^{-06}$	2.8 × $10^{-40}$	1.3 × $10^{-03}$
SB5	1.0 × $10^{-04}$	6.3 × $10^{-02}$	9.3 × $10^{-06}$	2.2 × $10^{-34}$	2.4 × $10^{-03}$
SB6	1.1 × $10^{-02}$	7.1 × $10^{-04}$	4.6 × $10^{-02}$	3.2 × $10^{-03}$	7.5 × $10^{-03}$

**Table 4:** Accuracy of SBs using multiple kernels of LS-SVM classifier.

Kernels	SB1	SB2	SB3	SB4	SB5	SB6
Linear	69.78	70.67	71.11	72.89	73.56	61.56
Polynomial	89.11	88	86.22	89.78	87.33	74.44
Mexican hat	86.89	84.22	91.78	92.44	89.78	64.67
Morlet	94	93.56	97.56	94.44	92.67	69.78
RBF	95.56	98.22	96	95.78	99.78	72.44

**Table 5:** Performance Parameter for channel 1 using RBF kernel.

Subband	ACC	SEN	SPE	F1-Score	MCC
SB1	95.56	97.13	93.69	0.960	91.05
SB2	98.22	98.02	98.48	0.984	96.40
SB3	96	97.15	94.60	0.963	91.93
SB4	95.56	97.53	93.71	0.961	91.52
SB5	99.78	99.60	100	0.998	99.55
SB6	72.44	75	69.19	0.752	44.14

**Table 6:** Confusion matrix of RF and RH.

Class	RF	RH
RF	100	0
RH	0.5	99.5

**Table 7:** Performance comparison with SOTA on the same dataset.

Articles	Method	Channel	Classifier	Feature	ACC
[30]	CWT	–	CNN	–	99.35
[27]	EMD	10	LS-SVM	5	97.56
[26]	TQWT	5	LS-SVM	4	96.89
[10]	FRCSP	18	EC	2	86.23
[23]	WPD	3	k-NN	6	92.80
[19]	OA	118	NB	11	96.32
[18]	OA	118	LS-SVM	11	96.62
[17]	M-CC	118	LR	6	93.10
[33]	DWT	118	LS-SVM	9	96.10
[32]	Z-score	–	LDA	6	81.10
[16]	CC	118	LR	–	91.79
[15]	CC	118	LS-SVM	6	95.72
[34]	CT	118	LS-SVM	9	88.32
[9]	R-CSP	1	R-CSP	6	83.90
[35]	CS	3	SVM	8	96.00
[38]	MSPCA-WPD	3	RF	6	98.69
[25]	RDWT	10	QSVM	5	99.11
Proposed method	r-TQWT	1	LS-SVM	5	99.78

## Data Availability

The data is taken from BCI Competition III which is available at https://www.bbci.de/competition/iii/(accessed on 31 December 2020).

## Abbreviations

The following abbreviations are used in this manuscript:

EEG	Electroencephalogram
MI	Motor imagery
TF	Time-frequency
FFT	Fast Fourier transform
R-CSP	Regularized common spatial patterns
FRCSP	Filtered regularized CSP
PSO	Particle swarm optimization
LS-SVM	support vector machine with least square
M-CC	Modified cross-correlation
OA	Optimal allocation
PLV	Phase lock value
NB	Naive Bayes
WT	Wavelet transform
EMD	Empirical mode decomposition
DWT	Discrete WT
RDWT	Rational dilation WT
TQWT	Tunable Q WT
CNN	convolutional neural networks
WPD	wavelet packet decomposition
MSPCA	multiscale principal component analysis
r-TQWT	Robust Tunable Q Wavelet Transform
SB	Subbands
LPS	lowpass subbands
HPS	High pass subbands
EOA	evolutionary optimization algorithms
SOTA	state-of-the-art techniques
ABC	Artificial Bee Colony
CS	Cuckoo search
